# Molecular Portrait of Autoantigens in Type 1 Diabetes

**DOI:** 10.3390/biom15121723

**Published:** 2025-12-11

**Authors:** Ilya Kandinov, Anastasia Knyazeva, Elizaveta Lander, Dmitry Gryadunov, Elena Savvateeva

**Affiliations:** Center for Precision Genetic Technologies for Medicine, Engelhardt Institute of Molecular Biology, Russian Academy of Sciences, 32 Vavilov Street, 119991 Moscow, Russia; aknyazeva04@gmail.com (A.K.); elleylander@gmail.com (E.L.); grad@biochip.ru (D.G.); len.savv@biochip.ru (E.S.)

**Keywords:** type 1 diabetes, autoantigens, β-cell, autoantibodies, epitope, multiplex diagnostics, immunotherapy

## Abstract

This review focuses on the molecular pathogenesis of Type 1 diabetes (T1D), specifically on the key autoantigens targeted by the autoimmune response and the clinical implications of their epitope specificity. T1D is characterized by the destruction of insulin-producing pancreatic β-cells. The autoimmune attack is directed against a defined set of autoantigens, primarily insulin, glutamic acid decarboxylase 65, tyrosine phosphatase-like protein, zinc transporter 8, as well as several minor autoantigens. A critical advancement in understanding the disease has been the analysis of epitope specificity, revealing that immunodominant epitopes are conformational and often localized to C-terminal protein regions, exposed during β-cell degradation. The introduction of sensitive multiplex assays for the simultaneous detection of T1D-associated autoantibodies represents a major diagnostic breakthrough. These platforms enable early diagnosis, risk stratification, and the identification of a “therapeutic window” for intervention. At this preclinical stage, antigen-specific immunotherapies aimed at restoring immune tolerance show significant promise. Ultimately, the combination of personalized diagnostic profiles, epitope mapping, and targeted therapies forms the basis for a new T1D management paradigm focused on halting the autoimmune process itself and preserving functional β-cell mass.

## 1. Introduction

Type 1 diabetes mellitus (T1D) is a chronic organ-specific autoimmune disease, characterized by the immune-mediated destruction of insulin-producing pancreatic β-cells [1,2]. This process, driven by a combination of genetic predisposition and environmental factors, results in absolute insulin deficiency. The consequent hyperglycemia necessitates lifelong insulin replacement therapy and continuous monitoring of glycemic status to prevent acute and chronic disease complications [3].

The epidemiology of T1D is characterized by significant geographical variations and a steady increase in incidence worldwide, posing a significant medical and social challenge [4]. The incidence rate ranges from approximately 1 case per 100,000 children per year in parts of Asia and South America to over 40–45 cases per 100,000 children per year in Northern European countries [5]. The annual global increase in incidence is most notable in young children, suggesting a strengthening impact of exogenous factors that trigger the autoimmune process. As this trend cannot be explained by genetic predisposition alone, it underscores the complex interplay between genetic susceptibility and environmental factors in the etiology of T1D [4,6].

Genetic factors play a significant role in the pathogenesis of T1D. The greatest contribution comes from genes of the major histocompatibility complex (MHC) class II. Specifically, the DR3-DQ2 and DR4-DQ8 haplotypes facilitate the more efficient presentation of autoantigenic peptides to CD4+ T-lymphocytes compared to other alleles, thereby initiating the pathological autoimmune cascade [7]. However, these haplotypes are neither necessary nor sufficient for disease development, underscoring the complexity of genetic predisposition [8]. Beyond the MHC locus, genome-wide association studies have identified additional susceptibility loci, including the insulin gene regulatory region and immune regulatory genes *CTLA-4* and *PTPN22* [9,10]. Although their individual effects are modest, these non-HLA genes collectively confirm the polygenic architecture of T1D and provide valuable insights for elucidating disease mechanisms.

Despite extensive research, the precise contribution of exogenous factors in triggering β-cell autoimmunity remains incompletely understood. Proposed environmental triggers include viral infections, co-infections, dietary factors (such as early introduction of cow’s milk or gluten and vitamin D deficiency), gut microbiota dysbiosis, and other lifestyle components [11,12,13]. These factors are thought to initiate the autoimmune process through several mechanisms, including molecular mimicry, impaired intestinal barrier function, systemic inflammation, or direct β-cell damage [14,15]. Ultimately, this cascade of events results in the exposure of previously sequestered autoantigens and the development of a pathological immune response.

A key event in the pathogenesis of T1D is the loss of immune tolerance to self-antigens of β-cells, leading to the activation of both humoral and cellular adaptive immunity. Autoreactive B-lymphocytes contribute to this process both as antigen-presenting cells and as sources of proinflammatory cytokines [16]. Furthermore, they produce high-affinity circulating autoantibodies (AAbs). While these AAbs are primarily considered disease biomarkers rather than direct pathogenic factors, they serve as crucial serological markers for identifying the preclinical stage of T1D [17,18].

The destruction of insulin-producing β-cells in T1D is executed by two primary immune cell populations. Cytotoxic CD8+ T-lymphocytes directly recognize and eliminate target cells by inducing programmed cell death [19]. Concurrently, CD4+ T-helper cells orchestrate the autoimmune response by activating CD8+ T-lymphocytes and secreting proinflammatory molecules that create a damaging inflammatory milieu around the islets of Langerhans [20].

Clinical manifestation of the disease occurs only after the destruction of 40–95% of the functional β-cell mass [2]. This prolonged preclinical stage, lasting from several months to several years, is characterized by the persistent presence of multiple autoantibodies and a progressive decline in pancreatic secretory function. This phase represents a critical “therapeutic window” for early disease diagnosis and for initiating interventions aimed at preserving residual β-cell mass [21].

The progression of the autoimmune response is driven by the recognition of specific molecular targets or autoantigens. Notably, the immune reaction is directed not against entire molecules, but against short, specific segments known as epitopes. The study of this epitope specificity, its temporal evolution (a phenomenon known as epitope spreading), and its correlation with clinical onset is fundamental to understanding the initiation and development of the autoimmune process [22].

The primary clinically significant autoantigens in T1D are insulin, glutamic acid decarboxylase (GAD65), tyrosine phosphatase-like protein (IA-2/ICA512), and zinc transporter 8 (ZnT8) [23]. Detection of high-affinity autoantibodies to these targets represents the “gold standard” for serological diagnosis and risk assessment [24,25,26,27]. Beyond these, a number of minor autoantigens are known (e.g., tetraspanin-7, ICA-69, IA-2β), though their clinical significance requires further clarification [28]. Therefore, a detailed investigation of the molecular properties and epitopes of these autoantigens is fundamental for developing novel tools for early diagnosis, prognosis, and personalized therapy.

This review aims to systematize current scientific data on autoantigens and their immunodominant epitopes in type 1 diabetes. It provides a comprehensive synthesis of the structural and functional characteristics of major and minor autoantigens and delineates their physiological roles within pancreatic β-cells. Furthermore, the review considers the potential applications of this knowledge for developing novel approaches to diagnosis, risk prediction, and immunotherapy for the disease.

## 2. Major Autoantigens

The development of serological diagnostics for T1D has paralleled the chronological discovery of its key autoantigens. The first biomarker, autoantibodies to insulin (IAA), was identified in the late 1960s [29]. This was subsequently followed in the early 1990s by the discovery of autoantibodies to glutamate decarboxylase (GAD65) [30,31]. The mid-1990s marked the next stage with the detection of autoantibodies to the pseudotyrosine phosphatase IA-2, a finding that significantly enhanced diagnostic specificity [32,33]. The modern diagnostic panel was completed in 2007 with the discovery of autoantibodies to zinc transporter 8 (ZnT8) [34]. Today, the simultaneous analysis of this panel—comprising autoantibodies to insulin, GAD65, IA-2, and ZnT8—constitutes the gold standard for serological diagnosis, providing optimal sensitivity and specificity for confirming the autoimmune etiology of T1D and for assessing disease risk [35].

The combined testing for all four autoantibodies exhibits a sensitivity of 95–98% and a specificity of 90–95% in newly diagnosed individuals, minimizing the likelihood of false-positive results [35]. This method serves as an indispensable tool for the presymptomatic diagnosis of T1D, even prior to clinical manifestation. In apparently healthy individuals, the presence of two or more autoantibodies is associated with a lifetime risk of developing T1D that exceeds 90% within the subsequent few years [17].

**Figure 1:**
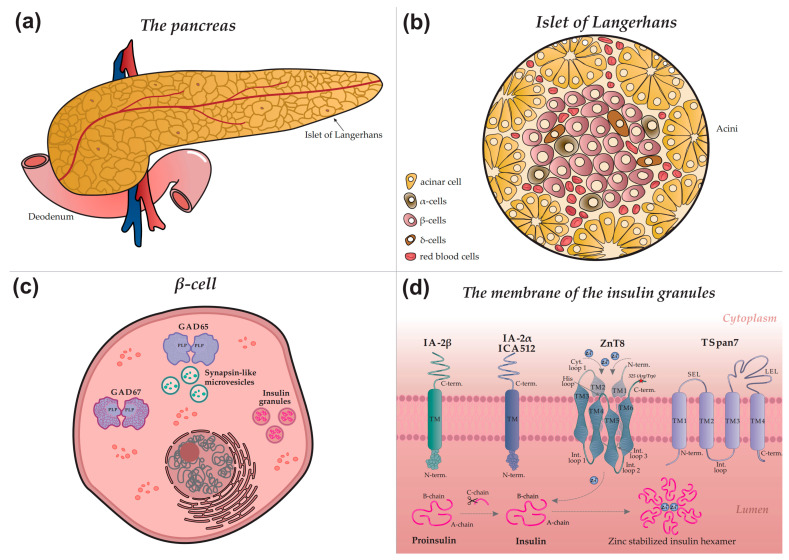
Autoimmune targets in type 1 diabetes: from organ to molecular structure (**a**) Schematic diagram of the pancreas. Surface nodules correspond to the islets of Langerhans. (**b**) Schematic structure of a pancreatic islet, indicating the main endocrine cell types: acinar, α—(alpha), β—(beta), δ—(delta) cells, as well as blood vessel cells. (**c**) Intracellular structure of a β-cell. Insulin granules, microvesicles, and the glutamic acid decarboxylase proteins GAD65 and GAD67 are shown. PLP, pyridoxal-5-phosphate. (**d**) Detailed view of an insulin granule membrane. Depicted autoantigens include IA-2β (phogrin), IA-2/IA-2α (ICA512, tyrosine phosphatase-like protein), ZnT8 (zinc transporter 8), and TSpan7 (tetraspanin 7), with their structural domains indicated. Proinsulin and insulin are present in the luminal space. Grey arrows denote the insulin maturation process, a key stage of which is ZnT8-mediated transport of zinc ions (Zn^2+^) into the granule, essential for the formation of mature hexameric insulin.

A comprehensive understanding of the mechanisms behind the autoimmune response and the generation of specific autoantibodies relies on elucidating the intracellular localization of autoantigens. Figure 1 provides a schematic overview illustrating the architecture of the pancreas (a), an islet of Langerhans (b), a β-cell (c), and the precise intracellular localization of major and minor autoantigens within the insulin granule (d).

### 2.1. Insulin

Insulin, a key regulator of glucose metabolism, is stored in pancreatic β-cells within secretory granules, where it is packaged into stable hexamers (Figure 1d) that dissociate into active monomers upon secretion. Its biosynthesis involves a multi-step pathway: preproinsulin, synthesized in the endoplasmic reticulum, is converted to proinsulin, which is subsequently cleaved during granule maturation to yield mature insulin and C-peptide [36,37,38]. The secretion of insulin into the bloodstream occurs via glucose-stimulated exocytosis, a process triggered by elevated blood glucose levels and a consequent rise in intracellular calcium concentration.

Current models propose that the autoimmune response against insulin in T1D develops through two principal mechanisms that disrupt natural immune tolerance [39]. The first involves rapid, massive β-cell destruction, which floods the bloodstream with large quantities of insulin and its precursors. This sudden antigen exposure leads to an overt breach of immune tolerance, whereby antigen-presenting cells capture proinsulin, insulin, and C-peptide, facilitating the presentation of previously cryptic epitopes and initiating an immune response [40]. The second mechanism is driven by chronic cellular stress, which disrupts autophagic and crinophagic pathways within β-cells. This dysregulation promotes the formation of modified protein structures, notably hybrid insulin peptides (HIPs), that are recognized by the immune system as foreign [41]. Although initiated by distinct triggers, both pathways ultimately converge on the activation of autoreactive T-lymphocytes, autoantibody production, and progressive β-cell loss.

**Figure 2:**
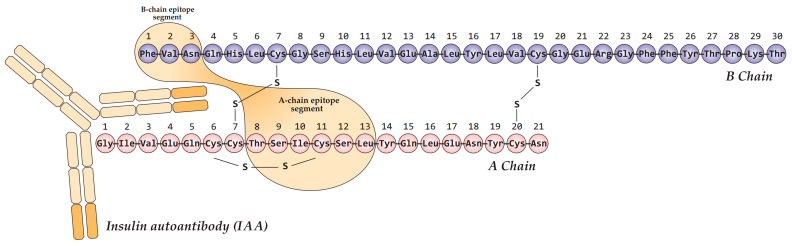
Schematic representation of the primary conformational epitope of insulin, formed by regions of the A-chain (A8–A13) and B-chain (B1–B3) in complex with an IAA autoantibody. Created based on [46].

Autoantibodies to insulin (IAA) associated with T1D primarily target conformational epitopes presented by the monomeric form of the hormone [42,43]. Multiple such epitopes have been reported, often exhibiting partial overlap and minor variations that likely reflect methodological differences across studies. The most extensively characterized immunodominant epitope is formed by the A-chain (residues A8–A13) and B-chain (B1–B3) of mature insulin (Figure 2) [44,45,46]. Therefore, the specific tertiary structure of insulin is critical for IAA recognition in T1D, highlighting the importance of conformational epitopes over discrete linear sequences.

A novel class of insulin epitopes, termed neoepitopes, has been identified in recent years. Their generation is linked to the unique biochemical environment of insulin secretory granules, which promotes the formation of hybrid insulin peptides [47,48]. These unique compounds are created through the covalent fusion of insulin fragments with other granule peptides, such as chromogranin A (forming HIP 2.5) or amylin (forming HIP 6.9) [49]. The resulting modified structures exhibit enhanced immunogenicity and are believed to play a pivotal role in both initiating and maintaining the autoimmune process, as they are recognized by the immune system as foreign entities.

### 2.2. Glutamic Acid Decarboxylase 65 (GAD65)

**Figure 3:**
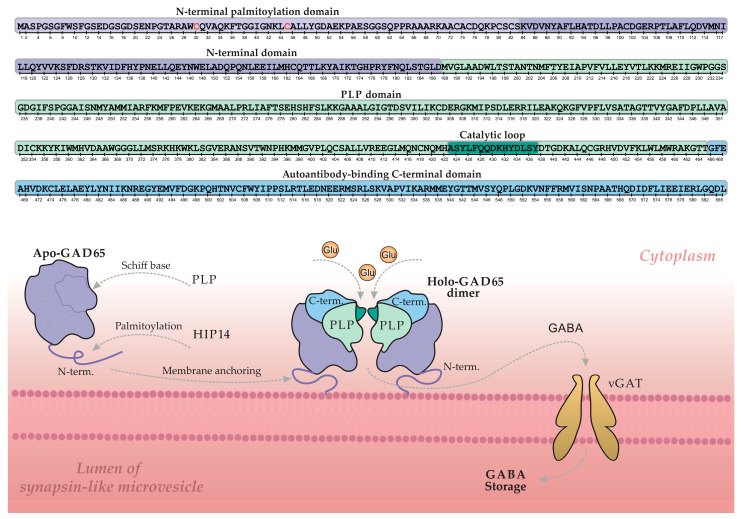
The regulatory cycle of GAD65 and GABA synthesis on the membrane of synapse-like microvesicles in a pancreatic β-cell. The upper section shows a linear schematic of the GAD65 amino acid sequence, with color-coded structural domains and key elements corresponding to the three-dimensional model below. Grey arrows indicate the principal stages of the cycle: palmitoylation, membrane anchoring, PLP cofactor binding, GABA synthesis, and GABA transport into the vesicle lumen via the vesicular GABA transporter (vGAT). Key cysteine residues (Cys30, Cys45) essential for palmitoylation are highlighted in red. Abbreviations: GAD, glutamic acid decarboxylase; PLP, pyridoxal 5′-phosphate; HIP14, huntingtin-interacting protein 14; GABA, γ-aminobutyric acid; Glu, glutamate. Adapted from [51].

Glutamic acid decarboxylase 65 (GAD65) is a key enzyme in pancreatic β-cells, catalyzing the synthesis of the primary inhibitory neurotransmitter γ-aminobutyric acid (GABA) from glutamate [50,51]. Its activity is dependent on the cofactor pyridoxal-5′-phosphate (PLP) [52]. Figure 3 illustrates the GABA synthesis cycle involving GAD65 within a β-cell, depicting the enzyme’s dynamic transition between its inactive, cytoplasmic apo-form (lacking PLP) and its active, membrane-bound holo-form (a PLP-bound homodimer).

The GAD65 molecule comprises three structural domains: an N-terminal domain, a PLP-binding domain, and a C-terminal domain. The N-terminal domain contains palmitoylation sites (Cys30 and Cys45) that, upon modification, serve as a hydrophobic membrane anchor. This palmitoylation is catalyzed by the palmitoyltransferase HIP14 (ZDHHC17), as illustrated in Figure 3 [53,54]. Following palmitoylation, GAD65 is firmly anchored to the membrane of synapse-like microvesicles within the β-cell, where it converts to the active holo-form and facilitates local GABA synthesis. Newly synthesized GABA is then transported into the vesicular lumen via the vesicular GABA transporter (vGAT) for storage and subsequent release. Regulation of enzymatic activity and GABA output is mediated by a catalytic loop within the PLP-binding domain [55,56].

The C-terminal domain of GAD65 is of particular interest, as it constitutes an immunological “hotspot” in T1D and represents the primary target for GAD65 autoantibodies (GADAs) [57,58,59]. GADAs predominantly recognize conformational epitopes dependent on the protein’s three-dimensional structure, and less commonly target linear epitopes. The spectrum of GAD65 epitopes is notably broad and demonstrates considerable heterogeneity across patients [60,61]. Reported variations in GADA epitope specificity may also reflect methodological differences and diversity in the patient cohorts studied, highlighting the importance of standardized approaches for consistent epitope mapping.

The principal, well-characterized immunodominant region of GAD65, located within the C-terminal domain (approximately residues 466–585), is highlighted in blue in Figure 3 (C-term) [62]. A second, less frequently targeted immunogenic region encompasses the segment between the C-terminal and PLP-binding domains (approx. residues 244–450). Notably, key conformational epitopes often depend on the spatial proximity between these two domains, an interaction particularly stabilized in the dimeric form of the protein. This underscores the critical role of GAD65′s tertiary and quaternary structure in its recognition by autoantibodies [52].

The N-terminal region of GAD65 (Figure 3), while not a direct target for most autoantibodies in T1D, is critical for the proper formation of conformational epitopes elsewhere in the molecule [63]. Intriguingly, studies demonstrate that an N-terminally truncated GAD65 isoform exhibits enhanced and more specific binding to T1D autoantibodies [64,65,66]. The structural mechanisms underlying this enhanced immunoreactivity remain to be fully elucidated and warrant further investigation.

In summary, the immune response against GAD65 in T1D exhibits complex topography, targeting a broad spectrum of epitopes. A detailed understanding of GAD65 autoantibody epitope specificity and its clinical implications remains a critical research goal. Although these autoantibodies are also present in other conditions like autoimmune polyglandular syndrome type I (APS-1) and stiff-person syndrome, their epitope profiles can differ substantially from those in T1D [67]. This disparity highlights the importance of precise epitope mapping for GAD65, not only to clarify the pathogenesis of T1D but also to elucidate the distinct autoimmune mechanisms operating across different diseases.

### 2.3. Tyrosine Phosphatase-like Protein (IA-2/ICA512)

IA-2 (Islet Antigen 2), also known as ICA512, is localized to the membrane of insulin secretory granules in β-cells (Figure 1d). Although it belongs to the receptor-like protein tyrosine phosphatase (PTP) family, IA-2 is classified as a pseudophosphatase, having lost its catalytic activity during evolution due to key amino acid substitutions in its active site while retaining the characteristic structural organization of the family [68,69]. The exact function of IA-2 is still being elucidated; however, it has been identified as a key regulator of insulin granule stability and pool size. Its absence accelerates granule degradation by enhancing autophagy and crinophagy. Furthermore, IA-2 plays a role in regulating the exocytosis of insulin-secreting granules, highlighting its critical role in insulin secretion mechanisms [70,71].

The molecular structure of IA-2 includes a cleavable signal peptide and three principal domains: an N-terminal domain oriented toward the insulin granule lumen, a transmembrane (TM) domain, and a large cytoplasmic C-terminal domain that houses the pseudophosphatase segment (Figure 1d) [23]. The immunodominance of the cytoplasmic domain contrasts with the lack of autoimmunogenicity observed in the N-terminal luminal domain. Accordingly, autoantibodies recognizing N-terminal epitopes of IA-2 are either absent or present only at very low frequencies in T1D patients. This is likely a consequence of the domain’s sequestration within the granule lumen, which limits its exposure to the immune system [72].

The cytoplasmic domain contains two immunodominant regions (aa 604–776 and 771–979), where major epitopes have been discovered [73]. Autoantibodies in T1D patients predominantly target the 771–979 region, a preference attributed to its unique tertiary structure stabilized by disulfide bridges among five conserved cysteine residues (C839, C849, C909, C919, C941) [74]. Thus, these disulfide bonds are essential for both structural integrity and the formation of a key conformational epitope. This specific architecture may facilitate cross-reactivity with homologous tyrosine phosphatases, thereby expanding the range of potential autoimmune targets in T1D—a phenomenon that constitutes an active research focus [75]. Another autoantigen from the tyrosine phosphatase family, IA-2β (phogrin), classified as minor, will be discussed in Section 3.2.

### 2.4. Zinc Transporter 8 (ZnT8)

Zinc transporter 8 (ZnT8), encoded by the *SLC30A8* gene, is a member of the SLC30 family and is predominantly expressed in pancreatic β-cells, where it localizes to the membrane of secretory insulin granules (Figure 1d) [76]. Its primary function is to actively transport zinc ions (Zn^2+^) from the cytosol into the granule lumen. The accumulated zinc plays a critical structural role by promoting insulin packaging: two zinc ions coordinate six insulin monomers to form a stable hexameric complex. This organization is essential for maintaining insulin’s conformational stability and enables its regulated secretion in response to elevated blood glucose levels [77,78,79].

The molecular structure of ZnT8 is characterized by homodimerization on the secretory granule membrane, a prerequisite for zinc transport. Each monomer integrates into the lipid bilayer via six transmembrane domains (TM1–TM6), with both N- and C-termini facing the cytosol (Figure 1d). The protein’s architecture features five connecting loops: three intragranular (IL1–IL3) and two cytoplasmic, including a short loop and a histidine-rich loop. The latter, situated between TM4 and TM5, is a key functional element that directly coordinates zinc ions [80]. Particular interest lies in the C-terminal domain, whose unique structure and surface accessibility establish it as the primary immunodominant epitope in T1D.

Autoantibody specificity against the C-terminal region of ZnT8 is determined by a single nucleotide polymorphism (rs13266634) in the *SLC30A8* gene (Figure 1d; Arg325Trp polymorphism indicated by a red star). The amino acid substitution of arginine for tryptophan at position 325 gives rise to two principal autoantigen variants: ZnT8-Arg (encoded by the C allele) and ZnT8-Trp (encoded by the T allele). The autoimmune response in T1D exhibits a striking allele-specific pattern; approximately 97% of C-allele carriers possess antibodies directed against ZnT8-325Arg, whereas T-allele carriers typically target ZnT8-325Trp [81,82]. Consequently, this high allelic specificity necessitates the inclusion of antigens corresponding to both variants in diagnostic panels to achieve optimal diagnostic sensitivity.

## 3. Minor Autoantigens

### 3.1. Glutamic Acid Decarboxylase 67 (GAD67)

In contrast to the major autoantigen GAD65, GAD67 is a cytoplasmic isoform uniformly distributed in the β-cell cytosol and does not associate with vesicle membranes (Figure 1c). Unlike the reversibly regulated GAD65, GAD67 is constitutively active, predominantly existing as a PLP-bound holo-dimer responsible for maintaining basal GABA levels. The GAD67 molecule comprises an N-terminal dimerization domain, a PLP-binding catalytic domain, and a C-terminal domain. Although autoantibodies to GAD67 are detected in approximately 60–70% of T1D patients, they typically result from cross-reactivity with GAD65 due to high inter-isoenzyme homology, thus defining GAD67 as a minor autoantigen [51]. Despite its minor status, the detection of GAD67-specific antibodies (distinct from cross-reactive ones) could potentially help refine patient stratification in complex cases, though its routine clinical use is currently limited due to the dominant role of GAD65.

### 3.2. Phogrin (IA-2β)

IA-2β (phogrin), encoded by the *PTPRN2* gene, is a homolog of IA-2/ICA512 (PTPRN1). In contrast to IA-2, phogrin localizes not only to insulin granule membranes but also to secretory vesicles in other endocrine cell types. Its molecular structure comprises an N-terminal pseudocatalytic domain, homologous to tyrosine phosphatases but catalytically inactive, a transmembrane domain (TM), and a cytoplasmic C-terminal domain (C-term) (Figure 1d). The C-terminal domain, stabilized by disulfide bridges between conserved cysteine residues, serves as the primary target for autoantibodies. Autoantibodies to IA-2β are detected in approximately 40–60% of newly diagnosed T1D patients, typically in conjunction with anti-IA-2 antibodies—a finding attributable to their extensive structural homology. Consequently, IA-2β is classified as a minor autoantigen [83]. The high structural homology with IA-2 means that IA-2β autoantibodies rarely provide independent diagnostic information. However, their detection may be associated with a more aggressive autoimmune process, and their inclusion in expanded panels could potentially improve risk prediction in specific subsets of patients.

### 3.3. Tetraspanin 7 (TSpan7)

Tetraspanin-7 (TSpan7), a member of the tetraspanin family, is localized in β-cells primarily on insulin granule membranes and, to a lesser extent, on the plasma membrane. Identified recently as a T1D-associated autoantigen, data on the prevalence of corresponding autoantibodies remain limited. Anti-TSpan7 autoantibodies are detected relatively infrequently, in approximately 5–10% of newly diagnosed T1D patients, underscoring their status as a minor autoantigen [84]. Tetraspanins organize specialized membrane microdomains (tetraspanin networks) hypothesized to participate in intercellular adhesion and exocytosis regulation. The TSpan7 structure features short intracellular N- and C-termini, four transmembrane domains, and two extracellular loops—a small (SEL) and a large (LEL) loop (Figure 1d). The LEL contains six cysteine residues forming stabilizing disulfide bonds. Its complex tertiary structure and surface accessibility establish the LEL as the primary target for autoantibodies in T1D [85]. The detection of anti-TSpan7 autoantibodies, while infrequent, holds promise for improving T1D diagnosis. This is especially relevant for seronegative cases, where it could help identify individuals who would otherwise be missed by standard serological screening.

### 3.4. ICA1 (Islet Cell Autoantigen 1, ICA69)

Islet Cell Autoantigen 1 (ICA1, or ICA69) is a 69 kDa cytoplasmic protein expressed in pancreatic β-cells and other neuroendocrine tissues. It is uniformly distributed throughout the cytosol and associates with membranes of immature secretory granules in the Golgi region. A key structural feature is its N-terminal BAR domain, which mediates heterodimerization with the BAR domain of PICK1. This functional complex plays a critical role in insulin granule biogenesis and maturation [86]. Autoantibodies to ICA69 are detected in approximately 15–20% of newly diagnosed T1D patients, thus supporting its classification as a minor autoantigen [87]. Although autoantibodies to ICA69 are not currently clinically useful as a standalone biomarker due to their high heterogeneity among patients, their detection may provide valuable insights into early disease manifestations.

## 4. Current Diagnostic Methods and Therapeutic Prospects for T1D

**Figure 4:**
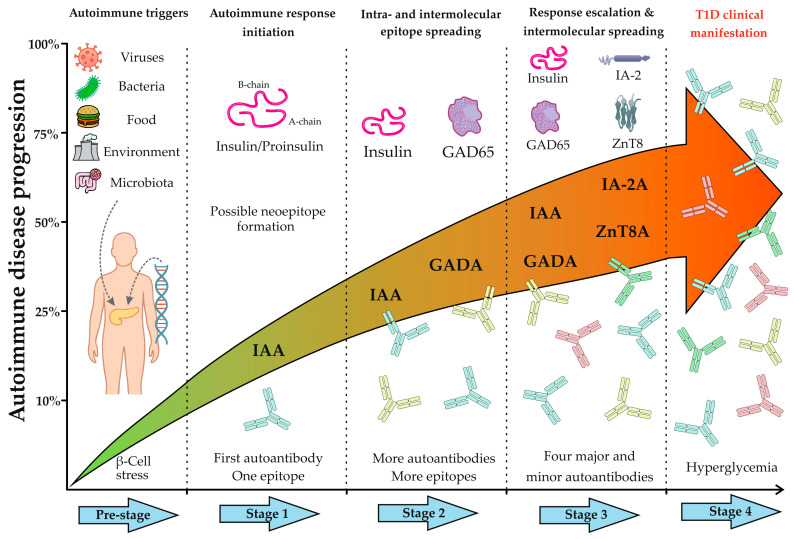
Timeline of autoimmune disease progression in Type 1 diabetes. The figure illustrates the key preclinical stages preceding the clinical onset of T1D. The Pre-stage depicts the initiation of autoimmunity in genetically susceptible individuals, triggered by exogenous factors (e.g., viruses, diet, environment, microbiota), leading to β-cell stress and immune activation. Stage 1 marks the beginning of the specific autoimmune response, characterized by early β-cell destruction and possible formation of neoepitopes or recognition by the immune system of one of the epitopes on beta cells. At this stage, patients typically test positive for a single autoantibody, most commonly against insulin or proinsulin (IAA), targeting a primary epitope. Stage 2 reflects the progression of the autoimmune response through sustained β-cell damage and intra- and intermolecular epitope spreading, resulting in a broader immune response, typically involving two autoantibodies (e.g., IAAs and GADAs). Stage 3 is defined by widespread intermolecular spreading and seropositivity for multiple autoantibodies against major (IAA, GADA, IA-2A, ZnT8A) and often minor antigens. Finally, Stage 4 represents the clinical manifestation of T1D, marked by symptomatic hyperglycemia due to a critical loss of functional β-cell mass.

Modern diagnostic approaches and therapeutic prospects for T1D are founded based on a thorough comprehension of its natural progression. The emergence of the autoimmune response in T1D follows a specific temporal sequence and hierarchy, reflecting the phenomenon of epitope spreading. The precise details of this process remain an area of active research and can vary based on patient demographics; however, studies have established that in young children, the immune response often commences with the appearance of autoantibodies to insulin (IAA), whereas individuals with later disease onset frequently develop autoantibodies to GAD65 (GADAs). Subsequently, as the autoimmune process progresses and β-cell destruction continues, the immune response broadens to include other antigens, such as IA-2 and ZnT8 [17,22]. Figure 4 outlines the fundamental stages of autoimmune progression.

Thus, Stages 1–3 (Figure 4) represent the most favorable “therapeutic window” for early diagnosis and the implementation of antigen-specific therapy (ASI). A key prerequisite for leveraging this “window” is the accurate determination of the disease stage, which is characterized by the dynamic shift in autoantibody profile. This evolution of the immune response, from a single autoantibody to a complex multi-autoantibody signature, necessitates a comprehensive diagnostic approach. Multiplex diagnostics fulfills this requirement by enabling the simultaneous capture of the complete serological profile in a single assay, thereby providing the data essential for effective risk stratification and informed clinical decision-making.

### 4.1. Multiplex Assays for the Detection of Diabetes-Associated Autoantibodies

The detection of preclinical T1D has undergone significant evolution in recent decades, marked by a paradigm shift from monoplex to multiplex platforms. These advanced systems enable a comprehensive profiling of the autoimmune response by simultaneously assessing antibodies against multiple autoantigens and their specific epitopes. This detailed epitope mapping provides crucial new insights into disease heterogeneity and progression, moving beyond simple antibody detection to characterize the fine specificity of the autoimmune process. This transition is driven by the clinical need for enhanced accuracy in early disease detection, improved risk stratification, and identification of concomitant autoimmune conditions. Furthermore, the ability to define individual epitope profiles holds significant potential for personalized risk assessment. The adoption of multiplex technologies thus represents a natural progression in the refinement of T1D diagnostics.

Traditional monoplex immunoassays—including radioimmunoassay (RIA), enzyme-linked immunosorbent assay (ELISA), and chemiluminescent immunoassay (CLIA)—remain the gold standard for confirming T1D autoimmunity and assessing disease risk [88]. These methods allow for the detection of autoantibodies against key antigens: insulin, GAD65, IA-2, and ZnT8 [28]. However, the requirement for separate analysis of each marker renders large-scale screening labor-intensive and costly, posing a significant limitation for comprehensive serological profiling. Although the prognostic value of a single autoantibody remains uncertain in asymptomatic individuals, the presence of two or more autoantibodies constitutes an unequivocal marker of high risk for progression to overt T1D.

#### 4.1.1. Array-ELISA

Array-ELISA represents a significant advance in T1D serodiagnostics, offering substantial improvements over conventional mono- and multiplex platforms [89]. This method enables the simultaneous yet discrete detection of five major autoantibodies (GADA, IA-2A, ICA, ZnT8-A, and IAA), thereby eliminating the requirement for supplementary confirmatory testing. A notable practical advantage is its compatibility with standard 96-well plates and routine laboratory instrumentation, ensuring accessibility for widespread clinical implementation. Consequently, Array-ELISA provides an optimal balance of high diagnostic sensitivity, specificity, and practical feasibility, making it particularly suitable for large-scale screening programs. A disadvantage of this method is that sorption of antigens, unlike covalent immobilization, can potentially lead to contamination of neighboring spots.

#### 4.1.2. Protein Microarrays

Protein microarray-based multiplex immunoassay represents a promising diagnostic approach for T1D, enabling simultaneous detection of a broad autoantibody profile. This encompasses both organ-specific antibodies (e.g., anti-GAD65, anti-IA-2, anti-ICA, anti-TPO, anti-TG, anti-21-OH) and markers specific for autoimmune polyglandular syndrome type I (e.g., anti-IFN-ω, anti-IFN-α2a, anti-IL-22) within a single assay [90]. Validation studies demonstrate high analytical reliability, with coefficients of concordance between the microarray platform and reference monoplex ELISA ranging from 0.75 to 0.92 for autoantibodies to GAD65, IA-2, and ICA. The overall agreement rate of 88–97% confirms the platform’s strong performance and suitability for comprehensive serological profiling. However, the method demonstrates lower sensitivity in head-to-head comparisons with established monoplex ELISA and requires further optimization.

#### 4.1.3. ADAP (Antibody Detection by Agglutination-PCR)

ADAP is a highly sensitive immunoassay that utilizes DNA barcoding technology. The technique involves covalently coupling T1D autoantigens or their epitopes to unique DNA oligonucleotides. Following incubation with a patient sample and specific binding to autoantibodies, these DNA tags are amplified by PCR, enabling exceptional detection sensitivity and specificity. This platform permits the simultaneous identification of autoantibodies against GAD65, IA-2, ZnT8, insulin, and thyroglobulin in a single reaction, making it a highly promising tool for large-scale screening of pediatric populations at elevated risk for T1D [91,92]. Nevertheless, this method remains experimental and has not yet been translated into routine clinical practice. Despite its outstanding sensitivity, the method remains experimental primarily due to its requirement for highly specialized equipment and reagents, high analysis costs, and the absence of standardized protocols.

#### 4.1.4. Phage Immunoprecipitation Sequencing (PhIP-Seq)

PhIP-Seq represents a powerful approach for multiplex serological profiling in T1D research [93]. This high-throughput technology immobilizes proteome-wide peptide libraries on a unified platform, enabling comprehensive characterization of antibody interactions within patient sera. The method facilitates global serological analysis by simultaneously screening thousands of peptide sequences representing diverse autoantigens [94]. While PhIP-Seq has proven invaluable for de novo autoantigen discovery, its primary limitation lies in preferential detection of antibodies targeting linear epitopes, making it less suitable for studying conformational epitopes that require intact tertiary protein structures. Furthermore, the method is characterized by complexity and high cost, requiring substantial computational resources for bioinformatic analysis, and thus remains primarily confined to research applications.

#### 4.1.5. Three-Screen Islet Cell Autoantibody Assay (ICA)

The Three-Screen ICA™ system (RSR Limited, Cardiff, UK) became one of the first commercially available solutions for the simultaneous detection of autoantibodies to GAD65, IA-2, and ZnT8 [95]. While it substantially accelerates analysis throughput, this method has a fundamental limitation: a positive result does not identify antibody specificity, necessitating confirmation with monoplex assays. Furthermore, the panel’s exclusion of insulin autoantibodies (IAA) reduces its diagnostic sensitivity, particularly for early-stage T1D and in pediatric populations. These constraints highlight the necessity for further development of this diagnostic approach.

#### 4.1.6. Multiplex Electrochemiluminescence (ECL)

Multiplex ECL immunoassays, pioneered by the research group of Liping Yu at the Barbara Davis Center for Diabetes (University of Colorado), constitute an advanced platform for T1D diagnostics [96]. This technology enables the simultaneous quantification of autoantibodies to insulin, GAD65, and IA-2, alongside antibodies to tissue transglutaminase (tTG), facilitating concurrent risk assessment for T1D and asymptomatic celiac disease [97]. The clinical utility of this multiplex ECL platform has been robustly validated in large-scale epidemiological studies, including the Autoimmunity Study in Kids (ASK), where it demonstrated high diagnostic accuracy and reproducibility in a cohort of 20,000 children [98]. The clinical implementation of this methodology is constrained by its dependency on specialized high-cost instrumentation, which presents a substantial barrier to widespread adoption in routine diagnostic settings.

In summary, contemporary T1D diagnostics are advancing towards integrated, multiparameter strategies that consolidate information from multiple autoantigens, their fragments, and epitopes. Established technologies such as protein microarrays, Three-Screen ICA, Array-ELISA, multiplex ECL, ADAP, and PhIP-Seq now provide the foundation for high-precision screening, early detection, and predictive risk assessment. These platforms facilitate the comprehensive detection of autoantibody repertoires while maintaining sensitivity and specificity comparable to traditional monoplex assays.

Despite these advances, persistent challenges include a lack of standardization, limited autoantigen panels, and the need for validation of novel biomarkers. A promising future direction involves generating personalized diagnostic profiles through epitope-specific mapping of individual immune responses—from full-length autoantigens to discrete epitopes. Such a strategy would unlock new potential not only for early T1D diagnosis but also for individualized prognosis and the development of targeted immunotherapies.

### 4.2. Antigen-Specific Immunotherapy (ASI)

In recent years, antigen-specific immunotherapy has emerged as a promising strategy aimed at the selective induction of regulatory T-lymphocytes (Tregs) and the restoration of immune tolerance to pancreatic β-cells. The primary targets for ASI in T1D are key autoantigens, including insulin, GAD65, IA-2, and ZnT8. A principal challenge in developing these therapies is the substantial heterogeneity of the autoimmune response among patients, requiring robust stratification strategies to achieve therapeutic efficacy.

The field of ASI is currently undergoing a pivotal transition from foundational mechanistic concepts to advanced clinical trials, facilitated by innovative delivery platforms and novel therapeutic strategies [99]. However, evaluating the clinical efficacy of various ASI modalities requires exceptionally complex and large-scale investigations, primarily due to the profound heterogeneity of autoimmune responses, taking advantage of the opportunity across patient populations. Current research focuses on developing and refining novel delivery systems for autoantigens or their immunodominant epitopes. Noteworthy examples include the use of gold nanoparticles [100] and biodegradable microgels designed for targeted antigen delivery and the induction of immune tolerance [101]. Furthermore, numerous ASI-focused clinical trials with published protocols are actively underway, evaluating diverse antigenic targets such as intranasal or oral insulin, GAD-alum, proinsulin-derived peptides, insulin B-chain constructs, and plasmid DNA vaccine (see Supplementary Table S1 for a consolidated overview). In parallel, the broader immunotherapy landscape in T1D also includes emerging modalities, such as CAR-T-cell therapy, antibody-based therapies, targeted cytokine modulation, microbiome-based interventions, and other novel immunotherapeutic strategies [102,103]. Collectively, these advancements herald a paradigm shift away from broad-spectrum immunosuppression toward the precisely targeted modulation of the autoimmune response, tailored to individual patient characteristics.

#### 4.2.1. Plasmid DNA Immunization

Plasmid DNA immunization represents a promising strategy for suppressing the autoimmune response against β-cell antigens while preserving systemic immune competence. This approach involves the in vivo delivery of plasmid vectors encoding target autoantigens or their immunodominant epitopes, facilitating endogenous synthesis of proteins in their native conformation. This enables antigen presentation under non-inflammatory conditions (“immune ignorance”), thereby promoting the induction of peripheral immune tolerance [99,104]. An advanced iteration of this strategy involves the co-expression of autoantigens with immunomodulatory cytokines such as IL-10. This engineered co-expression promotes the differentiation of naive T-cells into a regulatory phenotype (Treg) while concurrently inducing anergy in autoreactive effector T-lymphocytes [105].

#### 4.2.2. Peptide-Based Therapy

Peptide-based therapy employs synthetic fragments of autoantigens for precision modulation of the immune response. Unlike DNA immunization, which relies on endogenous antigen synthesis, this approach involves the exogenous administration of pre-formed peptides. The primary mechanism of action entails their uptake and presentation by immature dendritic cells in the absence of co-stimulatory signals, thereby promoting immune tolerance via the induction of regulatory T-cells and/or clonal deletion of autoreactive T-lymphocytes [106]. An alternative strategy utilizes altered peptide ligands (APLs): modified sequences with enhanced binding affinity for MHC class II molecules. These engineered ligands are designed to selectively stimulate regulatory T-cell populations rather than activating pathogenic effector responses [107].

#### 4.2.3. Intranasal Administration

Intranasal administration of autoantigens represents one of the most extensively investigated ASI approaches, exploiting the principle of mucosal tolerance induction [108]. Insulin has been the most thoroughly studied antigen in this context, with other T1D-associated antigens explored to a lesser extent. Following intranasal delivery, autoantigens are captured by specialized antigen-presenting cells in the nasal mucosa and the associated nasal-associated lymphoid tissue (NALT). This process triggers local activation and expansion of antigen-specific regulatory T-cells, which subsequently enter systemic circulation, migrate to the pancreas, and initiate restoration of immune tolerance directly at the site of autoimmune injury. Despite encouraging results in preclinical models and a favorable safety profile, large-scale clinical trials—including the Intranasal Insulin Trials (INIT I/II)—have not demonstrated significant efficacy in preventing T1D onset in at-risk children [109].

The therapeutic strategies discussed constitute promising approaches that nevertheless require consideration of heterogeneity in patient immune phenotypes, diversity of epitope specificity, variation in β-cell mass at the time of diagnosis, and challenges in identifying the optimal “therapeutic window”. Together, these limitations define the current obstacles that ASI must overcome before its full therapeutic potential can be realized. The success of such immunotherapy is contingent upon a specific “therapeutic window”, characterized by the presence of multiple autoantibodies alongside preserved functional β-cell mass. This patient population represents the primary target for ASI. Thus, the efficacy of antigen-specific therapy is directly dependent on early and precise diagnosis, underscoring the critical need for integrated programs that combine autoantibody screening, immune monitoring, and timely intervention. A personalized approach, tailored to individual autoantibody profiles and disease stage, represents a fundamental requirement for successfully re-establishing immune tolerance and maintaining residual insulin secretion.

## 5. Conclusions

Type 1 diabetes mellitus arises from a complex interplay of genetic susceptibility, immune dysregulation, and environmental triggers, culminating in the loss of immune tolerance and the destruction of pancreatic β-cells. The autoimmune response targets a well-defined set of β-cell autoantigens, with insulin, GAD65, IA-2, and ZnT8 constituting the primary targets.

In recent years, the analysis of autoantibody epitope specificity has proven crucial for elucidating T1D pathogenesis. It is now established that immunodominant epitopes for most of these autoantigens are conformational and often localized to C-terminal regions, which become exposed during β-cell degradation. Detailed epitope mapping has significantly advanced our understanding of the autoimmune process, particularly the phenomenon of epitope spreading, and helps delineate the progression from preclinical stages to clinical manifestation.

The introduction of modern, highly sensitive multiplex platforms for detecting T1D-associated autoantibodies into clinical practice provides a dual benefit: preventing acute complications like ketoacidosis and creating a unique opportunity for early diagnosis. This early detection opens a critical “therapeutic window” for intervention. At this preclinical stage, antigen-specific immunotherapy aimed at restoring tolerance to key autoantigens represents the most promising therapeutic strategy. The continued development of personalized diagnostic and therapeutic approaches, integrated with in-depth epitope mapping, is forging a new paradigm in T1D management. This strategy shifts the focus from managing insulin deficiency to actively halting the autoimmune process itself, with the ultimate goal of preserving a functional β-cell mass.

## Data Availability

Not applicable.

## Supplementary Materials

The following supporting information can be downloaded at: https://www.mdpi.com/article/10.3390/biom15121723/s1, Supplementary Table S1. Clinical Trials of Antigen-Specific Immunotherapies for Type 1 Diabetes.

## Abbreviations

The following abbreviations are used in this manuscript:

AAb	Autoantibody
ADAP	Antibody Detection by Agglutination-PCR
APLs	Altered Peptide Ligands
ASI	Antigen-Specific Immunotherapy
ASK	Autoimmunity Study in Kids
CLIA	Chemiluminescent Immunoassay
ECL	Electrochemiluminescence
ELISA	Enzyme-Linked Immunosorbent Assay
ENDIT	European Nicotinamide Diabetes Intervention Trial
GABA	Gamma-Aminobutyric Acid
GAD65	Glutamic Acid Decarboxylase 65
GAD67	Glutamic Acid Decarboxylase 67
GADA	GAD65 Autoantibodies
HLA	Human Leukocyte Antigen
HIP14	Huntingtin-Interacting Protein 14
IA-2	Islet Autoantigene 2
IAA	Insulin Autoantibodies
ICA1	Islet Cell Autoantigen 1
LEL	Large Extracellular Loop
MHC	Major Histocompatibility Complex
NALT	Nasal-Associated Lymphoid Tissue
PCR	Polymerase Chain Reaction
PhIP-Seq	Phage Immunoprecipitation Sequencing
PICK1	Protein Interacting with C Kinase 1
PLP	Pyridoxal-5′-phosphate
RIA	Radioimmunoassay
SEL	Small Extracellular Loop
T1D	Type 1 Diabetes
TM	Transmembrane Domain
Treg	Regulatory T-Lymphocytes
